# Mild blurry vision as the initial presentation of central nervous system relapses of acute lymphoblastic leukemia: a case report

**DOI:** 10.1186/s12886-022-02697-0

**Published:** 2022-12-22

**Authors:** Yuehong Zhang, Zhimeng Zhang, Wenjian Mo

**Affiliations:** 1 Department of Ophthalmology, Guangzhou First People’s Hospital, School of Medicine, South China University of Technology, Guangzhou, 510080 China; 2 Department of Hematology, Guangzhou First People’s Hospital, School of Medicine, South China University of Technology, Guangzhou, 510080 China

**Keywords:** Case report, Optic nerve edema, Hematopoietic stem cell transplantation, Leukemia relapses, Central nervous system

## Abstract

**Background:**

Leukemia relapses after hematopoietic stem cell transplantation can sometimes occur from the central nervous system prior to relapse from the bone marrow, and manifestations varied.

**Case report:**

We present a case of mild blurry vision as the initial symptom of central nervous system relapse of adult acute lymphoblastic leukemia. A 30-year-old man presented with a 1 week history of painless visual loss in both eyes. At that time there were no headaches or other systemic features. The neurological examination was without positive findings except bilateral optic nerve edema. He had a history of acute lymphoblastic leukemia and hematopoietic stem cell transplantation, which had been in clinical remission post-transplant for 1 year. Lumbar puncture revealed relapsed disease within the central nervous system, confirmed with cerebrospinal fluid leukemic blasts.

**Conclusions:**

It highlights the need for ophthalmologists to be aware of the possibility of central nervous system involvement in patients with the setting of leukemia when visual symptoms as the initial manifestation.

## Background

Central nervous system (CNS) relapses after hematopoietic stem cell transplantation (HSCT) from acute lymphocytic leukemia (ALL) is not unusual, but the mild blurry vision as the first and only symptom of relapse occurs infrequently. The most common site of ophthalmic involvement in ALL patients is the retina, while leukemic infiltration of the optic nerve is less reported, especially in adult patients [1, 2].

Leukemia relapse can sometimes occur from the CNS before relapse from the bone marrow. Some recurrent patients present with extramedullary manifestations even in the absence of trace of bone marrow relapse [3]. Patients with CNS involvement pre transplant have higher risk of CNS relapse [4]. The risk factors for CNS relapse in ALL patients also include T-cell ALL, male sex, the presence of BCR-ABL fusion in B-cell ALL, and presenting leukocyte count 50 × 10^9^/L in T-cell ALL [5]. Neither pre-transplant cranial irradiation nor the use of total body irradiation (TBI)-based conditioning, nor post-transplant prophylactic intrathecal chemotherapy, was associated with a reduction of CNS relapse risk post-transplant [6]. Moreover, criteria for CNS prophylaxis during transplantation have not been addressed, so the diagnosis and prevention of CNS relapse after HSCT remains a challenge. It’s usually not easy to identify CNS relapse until the manifestations of cranial nerve involvement appear such as headache, diplopia, dysarthria and palpebral ptosis.

Here we report an uncommon CNS relapse case of ALL presenting with bilateral optic nerve edema and vision loss in a HSCT recipient believed to be in systemic remission.

## Case report

**Fig. 1:**
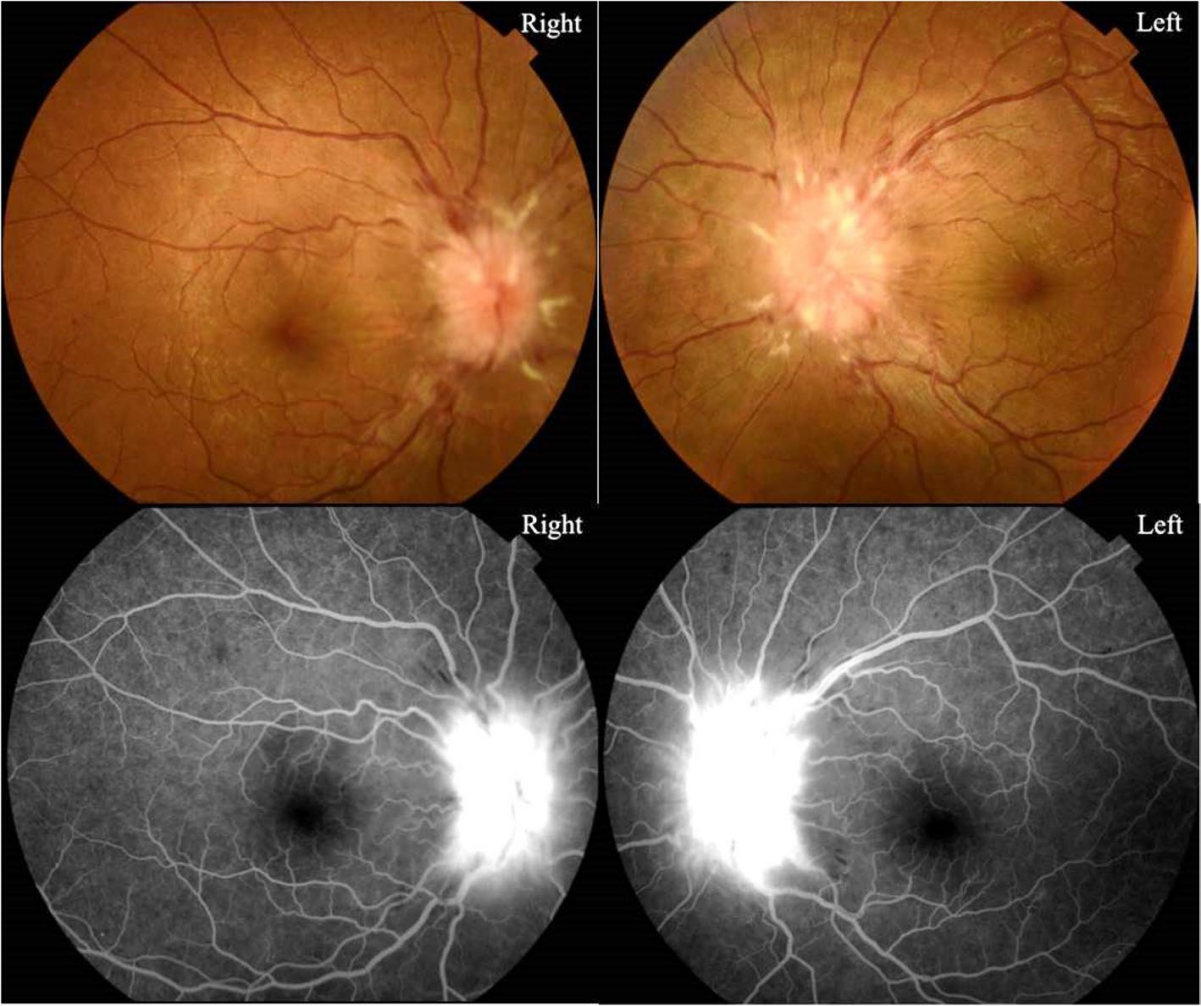
Fundus photograph of the both eyes showing significant bilateral optic nerve edema and linear peripapillary hemorrhages. Fundus fluorescein angiography of the both eyes showing hyperfluorescence in the optic disc

**Fig. 2:**
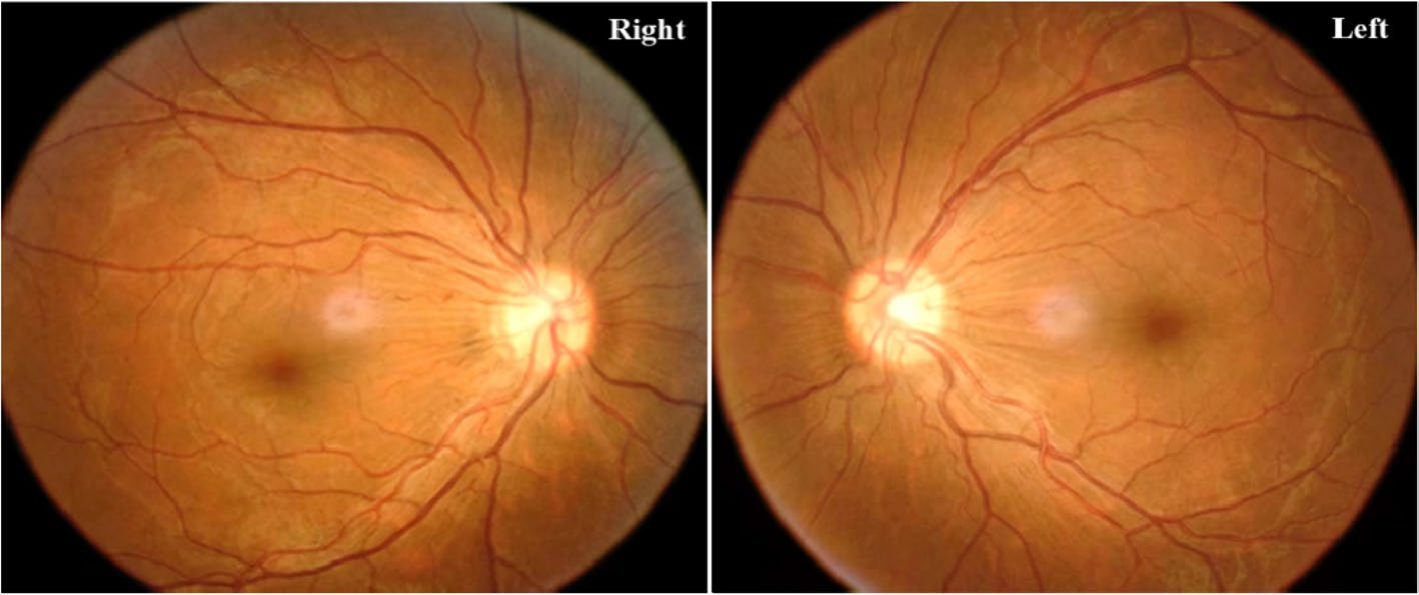
Fundus photograph of the both eyes showing reduction in optic nerve edema at the 22nd month of follow-up after central nervous system relapses of acute lymphoblastic leukemia

A 29-year-old male was first diagnosed with ALL in May 2019 when he presented with dizziness, fatigue and occasional blackness. At that time, hematological examination showed a hemoglobin level of 56 g/L (normal range: 120-140 g/L), a platelet count of 31 × 10^9^/L (normal range: 100–300 × 10^9^/L), and a white blood cell count of 285 × 10^9^/L (normal range: 8–10 × 10^9^/L) with 82.5% primary and juvenile lymphocytes. The phenotypes of leukemic blasts in bone marrow by flow cytometer were CD10+, CD19+, CD34+, CD45dim+, CD123 + and HLA-DR+. Chromosomal analysis showed that BCR/ABL fusion gene was positive and translocation occurred on chromosomes 9 and 22. Bone marrow aspirate and biopsy confirmed the diagnosis of Philadelphia chromosome-positive ALL. He achieved complete remission after the first induction chemotherapy and dasatinib administration as well as 3 courses of consolidation chemotherapy. He then underwent HLA-matched sibling allogeneic HSCT. The conditioning regiment was BUCy + VP16 (cytarabine 2 g/m^2^ for 2 days, Busulfan 0.8 mg/kg q6h for 3 days, cyclophosphamide 1.8 g/m^2^ for 2 days, etoposide 24 mg/kg for 2 days and anti-thymocyte immunoglobulin 2.5 mg/kg for 2 days) and prevention strategies for graft versus host disease included methotrexate and cyclosporine. He did not receive cranial irradiation. He had no CNS involvement pre-transplant and had received 5 times of intrathecal chemotherapy for CNS leukemia prophylaxis before HSCT. Dasatinib was also used to prevent leukemia relapse 1 month after transplantation, but it had to be stopped and replaced by imatinib because he developed moderate pleural effusion 1 month after oral administration of dasatinib. The patient had been arranged to bone marrow and peripheral blood examination for relapse of leukemia, as well as fundus examination for cytomegalovirus (CMV) retinitis due to his preceding CMV viremia after HSCT every month. He had remained asymptomatic without bone marrow or peripheral blood involvement or CMV retinitis after more than 1 year of follow-up. There was no prior history or evidence of ALL CNS involvement throughout his clinical course. A routine hematological examination 1 month before the onset of ocular symptom revealed no evidence of leukemic blasts by morphological and flow-cytometric analysis in bone marrow. In August 2020, in the course of a regular fundus examination for CMV retinitis, he complained of a mild blurry vision in both eyes with no headache or other systemic features. The visual acuity was 20/40 in each eye. Anterior segment examination was unremarkable in both eyes. Dilated fundus examination revealed significant optic nerve edema and linear peripapillary hemorrhages bilaterally (Fig. 1). CT brain demonstrated no abnormality. Laboratory investigations including complete blood count, liver and renal function tests and infected markers were normal. We performed lumbar puncture and the initial cerebrospinal fluid (CSF) pressure was 29 cm of water. CSF sampling was performed on suspicion of extramedullary recurrence of the disease, and revealed the abundant of leukemic blasts. The results of bone marrow smear showed that no naive cells were found and minimal residual lesions detected by flow cytometer and BCR-ABL/ABL (P190) fusion gene were both negative. This patient was diagnosed with recurrent ALL with isolated CNS relapse, and was treated immediately with nilotinib at a dose of 400 mg, twice a day. He also received cytarabine and methotrexate for intrathecal injection twice a week until no leukemic blasts were detected in CSF. Over the next 9 months, serial lumbar puncture test results showed an absence of blast cells. In June 2022, a recent ophthalmic examination showed that the patient’s vision had improved to 20/20 in each eye with reduction in optic nerve edema (Fig. 2).

## Discussion

There are several difficulties in the early diagnosis of this case. First, mild blurry vision as first and sole presentation is not the most common extramedullary manifestation of CNS involvement in ALL patients. Second, high intracranial pressure (ICP) is usually presented with papilledema and headache, while this patient had no headache. Third, analysis of leukemia blasts in CSF is not routinely performed unless there is clinical suspicion of CNS leukemia. The last, this patient has no risk factor of CNS involvement and had received intrathecal chemotherapy for CNS relapse prophylaxis before HSCT, which are easy to ignore the possibility of CNS involvement. Our case indicates that bilateral subacute visual loss with bilateral optic nerve edema may be the first indication of CNS relapse of ALL. The ophthalmologist plays a critical role in identifying leukemic ocular involvement, especially when a patient with a setting of leukemia is in clinical remission.

CNS relapse of ALL can be determined by the morphologic evidence of leukemic blasts in the CSF, manifestations of cranial nerve palsies or non-hemorrhagic mass induced by infiltration of leukemic cells in cranial imagines [4]. In this case, the differential diagnosis of bilateral optic nerve edema was the key to the definitive etiological factor. We first ruled out the possibility of intracranial mass lesion, malformation and ventriculomegaly. And then we excluded infections of the CNS, toxic effects of chemotherapeutic agents, or ischemia of the optic nerve. It is suspected that the optic nerve edema of this patient may be due to high ICP, so we performed lumbar puncture for him and added extra the detection of leukemic blasts and CMV in CSF in addition to the routing CSF test items. CSF analysis was negative for glucose and infectious agents; however, CSF sampling demonstrated leukemic CNS involvement.

Pui CH et al. [7] demonstrated that intensive chemotherapy including dasatinib could yield superior results in the treatment of Philadelphia chromosome-positive ALL compared with imatinib and provided excellent control of CNS leukemia without the use of prophylactic cranial irradiation. In this case, dasatinib combined with chemotherapy achieved good efficacy before transplantation, but after transplantation, the patient developed pleural effusion after administration of dasatinib and switched to imatinib. After CNS relapse, imatinib was replaced by nilotinib which is another second-generation Abl-tyrosine kinase inhibitor. After administration of nilotinib plus multiple intrathecal injections of chemotherapy, the patient regained CNS remission without significant side effects. CD19-directed engineered T cells expressing a chimeric antigen receptor (CAR-T) was also considered as an effective treatment for CNS relapse of ALL [8], but the patient refused the treatment option for financial reasons.

In this case, the diagnosis of CNS relapse case of ALL was made quickly based on the result of lumbar puncture, so we cancelled some additional examinations, such as visual field and brain MRI examination originally scheduled. This led us to be unable to clearly identify whether the optic nerve edema of this leukemia patient was due to increased ICP or direct infiltration of the optic nerve. The fact that the opening pressure was elevated on lumbar puncture would suggest high ICP. Papilledema induced by increased ICP usually does not cause a decrease in visual acuity unless there is optic atrophy, ischemic changes, or subretinal fluid tracking into the fovea. However, the visual acuity of this patient was decreased and the typical symptoms of high ICP (such as headache) were absent, which seems to be more consistent with direct infiltration. Direct infiltration of leukemic blasts within the eye is rare. When it does occur, it is related to the accumulation of circulating leukemic cells in the uvea. It can appear as leukemic pseudo-hypopyon or optic disc infiltration, such as a papilledema with peripapillary hemorrhages or white-grayish subretinal lesions due to choroidal infiltration which was absent from our case. However, without supportive testing, it is still hard to definitively differentiate the cause of optic nerve edema.

In summary, our case emphasized the importance of continued fundus examination of patients with a history of ALL and HSCT for signs of leukemic infiltration of the retina and optic nerve. This patient had been scheduled for fundus examination for CMV retinitis due to his preceding CMV viremia after HSCT every month, so the presence of optic nerve edema was noted. It is concluded that mild blurry vision may be the earliest manifestation of CNS relapses of ALL patients. Timey lumbar puncture to exclude the intracranial hypertension and the detection of leukemic blasts in CSF may facilitate early identification of CNS involvement in this population.

## Abbreviations

CNS: Central nervous system
ALL: Acute lymphoblastic leukemia
HSCT: Hematopoietic stem cell transplantation
CSF: Cerebrospinal fluid
CMV: Cytomegalovirus
ICP: Intracranial pressure
